# Coronary Artery Spasm: New Insights

**DOI:** 10.1155/2020/5894586

**Published:** 2020-05-14

**Authors:** Anthony Matta, Frederic Bouisset, Thibault Lhermusier, Fran Campelo-Parada, Meyer Elbaz, Didier Carrié, Jerome Roncalli

**Affiliations:** ^1^Department of Cardiology, Institute CARDIOMET, CHU-Toulouse, Toulouse, France; ^2^Faculty of Medicine, Holy Spirit University of Kaslik, Kaslik, Lebanon

## Abstract

Coronary artery spasm (CAS) defined by a severe reversible diffuse or focal vasoconstriction is the most common diagnosis among INOCA (ischemia with no obstructive coronary artery disease) patients irrespective to racial, genetic, and geographic variations. However, the prevalence of CAS tends to decrease in correlation with the increasing use of medicines such as calcium channel blockers, angiotensin converting enzyme inhibitor, and statins, the controlling management of atherosclerotic risk factors, and the decreased habitude to perform a functional reactivity test in highly active cardiac catheterization centers. A wide spectrum of clinical manifestations from silent disease to sudden cardiac death was attributed to this complex entity with unclear pathophysiology. Multiple mechanisms such as the autonomic nervous system, endothelial dysfunction, chronic inflammation, oxidative stress, and smooth muscle hypercontractility are involved. Regardless of the limited benefits proffered by the newly emerged cardiac imaging modalities, the provocative test remains the cornerstone diagnostic tool for CAS. It allows to reproduce CAS and to evaluate reactivity to nitrates. Different invasive and noninvasive therapeutic approaches are approved for the management of CAS. Long-acting nondihydropyridine calcium channel blockers are recommended for first line therapy. Invasive strategies such as PCI (percutaneous coronary intervention) and CABG (coronary artery bypass graft) have shown benefits in CAS with significant atherosclerotic lesions. Combination therapies are proposed for refractory cases.

## 1. Introduction

Coronary artery spasm (CAS), which is a reversible vasoconstriction driven by a spontaneous vascular smooth muscle hypercontractility and vascular wall hypertonicity narrowing the lumen of normal or atherosclerotic coronary arteries compromising the myocardial blood flow, is recognized recently under the chapter of myocardial infarction with nonobstructive coronary arteries (MINOCA) [1, 2]. Several features were attributed to this complex ischemic entity over time passing by “A variant form of angina pectoris or variant angina” [3], “variant of the variant” [4], “coronary vasospastic angina” [5], “a false-positive STEMI” [1], and “forgotten coronary disorder” [6].

The concept of CAS was first postulated by Prinzmetal et al. by describing a nonexertional angina occurring at rest or during regular daily activities [3], which could not be explained by an increase in myocardial oxygen demand unlike the classical angina of Heberden induced by an emotional or physical stress and relieved by exercise cessation or nitrates [7, 8]. Thus, they proposed an underlying culprit vasospasm reducing blood supply to a localized myocardial area [3] that explains the remarkable accompanied electrical changes such as transient ST segment elevation or depression in the corresponding leads [9, 10].

Recently, the coronary artery spasm hypothesis was confirmed and demonstrated in several experimental studies, especially after the introduction of either the provocative test that induces vasospasm [6, 11] or coronary angiography that illustrates spasm on the epicardial coronary artery in patients with vasospastic angina [4, 12, 13]. As a result, CAS acquired an essential contributing role in ischemic heart disease pathophysiology. This article is an update review of CAS, highlighting the unfamiliar subclinical entity known as “Kounis syndrome” and the latest development in the diagnostic modalities such as CMRI, IVUS, and OCT.

## 2. Epidemiology

CAS prevalence varies widely among races and between countries, but it remains the main cause of ischemic heart disease with nonobstructive coronary lesions [14]. It was estimated at 50% in patients presenting with angina and 57% in whom presenting with ACS [15–17]. In fact, CAS is more common in males than females [5, 18], individuals aged between 40 and 70 years [5, 18], and more in Japanese (24.3%) followed by Taiwanese (19.3%) and Caucasian (7.5%) populations [19]. The widespread use of calcium channel blockers, statins, angiotensin II receptor blockers, and converting enzyme inhibitors, smoking awareness campaigns, and declining tendency of physicians to carry out coronary vasoreactivity tests contribute to a reduction in CAS prevalence, particularly in Japan [20, 21].

## 3. Clinical Features

### 3.1. Symptoms and EKG

The length of the CAS episode is important in terms of the large variance of clinical manifestations from the asymptomatic event to the different aspects of ACS (unstable angina, NSTEMI, and STEMI) and sudden cardiac death [22–25]. Nonetheless, silent ischemia often found with a short episode of CAS is twice as prevalent as angina pectoris and chest pain, which is considered to be the most common feature related to CAS [20]. Therefore, a prolonged CAS accelerates the progression of atherosclerosis and triggers thrombus formation by platelets activation [14, 26]. Unlike vasovagal symptoms such as nausea, vomiting, and cold sweat, the circadian variability characteristic for CAS occurs regularly at rest and early in the morning between midnight and 5 a.m. and especially while performing light exercises [27, 28]. This is thought to be the result of the autonomous nervous system involvement in the pathophysiology of CAS [21, 29]. Furthermore, transient elevation of the ST segment occurs less often than the depression of the ST segment usually accompanying a spasm of a small coronary artery, a nondiffuse or nonsevere spasm of major coronary artery, and a spasm of major occluded coronary artery supplied by collaterals, which resume the main electrocardiographic modifications revealing CAS [9, 10, 30]. Also, various types of ventricular and supraventricular arrhythmias could be observed [20].

### 3.2. Kounis Syndrome

Kounis syndrome is an unfamiliar form of CAS appearing in a specific situation characterized by the simultaneous occurrence of ACS and hypersensitivity reaction. It is known as “allergic angina” or “allergic myocardial infarction” [31]. CAS constitutes the core component of the physiopathology of different forms of Kounis syndrome, particularly type 1 and 2, which are differentiated according to coronary angiography. Type 1 is a pure spasm involving a normal coronary artery, while type 2 is a spasm occurring on an atherosclerotic coronary artery causing plaque rupture. Variant inflammatory mediators such as histamines and leukotrienes massively secreted in the peripheral circulation during hypersensitivity reaction trigger CAS via their effects on the smooth muscle of coronary vessels [32]. Since Kounis syndrome is little known among physicians, we draw attention to this clinical form of CAS through this review knowing that an early appropriate approach is ultimately needed to avoid progression to a critical clinical situation [33].

## 4. Risk Factors and Precipitating Factors

### 4.1. Risk Factors

The main risk factors for CAS are age, smoking, hypertension, LDL-cholesterol, diabetes mellitus, and Hs-CRP [34–36]. Moreover, smoking has a great impact on CAS in male gender [18, 35], younger subjects [37], and Japanese population [38] as opposed to female gender, older individuals, and Caucasian population. The coexistence of several risk factors strengthens their effects to induce CAS, which may on itself sustain CAS leading to a vicious circle [23, 28]. However, diabetes mellitus is more potent in triggering CAS for women with lower Hs-CRP compared to those with higher levels of Hs-CRP [35, 39–41].

### 4.2. Precipitating Factors

A long-term mental stress [42], a light exercise especially in the early morning [28], cold exposure [43], hyperventilation [44, 45], magnesium deficiency [46], valsalva maneuver [44, 45], alcohol consumption [47], cocaine use [48], pharmacological sympathomimetic or parasympathomimetic or beta blocking or anticholinesterase agents [28], phentermine consumption [6], and platelets activation via vasoconstriction agents (thromboxane and serotonin) [49, 50] can precipitate CAS. As well, an alcohol ingestion following a stressful event can induce CAS within several hours [51, 52].

## 5. Pathophysiology

The exact pathophysiology of CAS is not clearly understood. However, CAS phenomenon is multifactorial implicating the autonomic nervous system, endothelial dysfunction, inflammation, oxidative stress, smooth muscle hyperreactivity, atherosclerosis, thrombosis, and genetic predisposition (Table 1).

### 5.1. Autonomic Nervous System

The complex relation between CAS and the autonomic nervous system is illustrated via the contribution of its two components: the sympathetic and the parasympathetic nervous system [19]. The role of the parasympathetic nervous system is supported by the frequent occurrence of CAS at midnight or at rest correlating with the highest vagal activity [53, 54] and by the ability of acetylcholine to induce CAS [55]. As such, the increased levels of catecholamines following an ischemic episode of CAS [56] and the occurrence of CAS at night during the rapid eye movement phase of sleep are marked by a reduction of vagal tone and increased adrenergic activity, enhancing the contribution of the sympathetic nervous system [57]. In fact, alpha blockers are not effective in controlling CAS symptoms [58]. On the other hand, nonselective beta-1 adrenorecptor blockers such as propranolol promote CAS as opposed to selective blockers such as atenolol [59, 60].

### 5.2. Endothelial Dysfunction

The nitric oxide (NO) produced by a normal functional endothelium enhances vasodilatation via suppressing the release of vasoconstrictors agents such as angiotensin II and endothelium I [61, 62]. Therefore, the deficiency in NO due to dysfunctional endothelial nitric oxide synthase explains the fact that different endothelium-dependent vasodilators such as ergonovine, histamine, acetylcholine, and serotonin, which are supposed to induce coronary vasodilatation, provoke vasoconstriction in patients with CAS [63, 64]. Also, it explains the high sensitivity of vasospastic angina to nitrate, an independent-endothelium vasodilator [65]. Meanwhile, endothelial dysfunction that may predispose to CAS is not present in all cases.

### 5.3. Chronic Inflammation

The relationship between inflammation and CAS was first described by Lewis et al. in a case report of variant angina and pericarditis [66]. An increased level of Hs-CRP, monocyte and WBC counts, interleukin-6, and adhesion proteins found in CAS patients are the culprits contributing the inflammatory mechanism [39, 67]. This correlation was observed; for example, the administration of interleukin-B triggers CAS [68]. In addition, chronic smoking is associated with chronic inflammation, which is the main risk factor for CAS [69]. Recently published studies suggest the inflammatory enrollment of the coronary vascular adventitia and adipose tissue [70].

### 5.4. Oxidative Stress

Oxygen free radicals stimulate vasoconstriction and endothelial damage via degrading nitric oxide released by endothelial cells [71]. The increased level of thioredoxin, a marker for oxygen reactive agents [72], in combination with reduced level of antioxidants such as vitamins C and E [73] support the role played by oxidative stress in CAS addressing the direct contribution of endothelial dysfunction in coronary spastic angina [74].

### 5.5. Smooth Muscle Hypercontractility

Phosphorylation and dephosphorylation of the myosin light chain (MLC) regulate coronary smooth muscle reactivity [75, 76]. In CAS, the increased activity of Rho-kinase sensitizes vascular smooth cells to Ca + enhancing MLC phosphorylation, which promote vasoconstriction [75]. Similarly, multiple pathways are involved in coronary smooth muscle cell hypercontractility such as nitric oxide [77], phospholipase C [78], and K_ATP_ channels [79, 80].

### 5.6. Atherosclerosis and Thrombosis

CAS and atherosclerosis are two distinct clinical entities that share common risk factors such as male gender, smoking, elevated Hs-CRP, dyslipidemia, and diabetes mellitus [34, 81]. Both entities may coexist, and the progression of either one influences the evolution of the other [23, 28]. In recent studies, it is shown that stent angioplasty for coronary lesions does not affect the recurrence of CAS that may happen in the distal parts of lesions [82–84]. In comparison with atherosclerosis, the prevalence of CAS tends to decrease with age. [20]. It is also well known that a prolonged CAS triggers thrombus formation through platelet activation [85] and mediator secretions such as fibrinopeptide A, which increases following a CAS event [86, 87].

### 5.7. Genetics

Genetic polymorphisms or mutations coding for angiotensin converting enzyme [88], paraoxonase I [89], adrenergic receptors [90], inflammatory mediators [78, 91], endothelial nitric oxide synthase [92, 93], and serotoninergic receptors [94] play a role in the pathogenesis of CAS. Studies identified some susceptible genes such as those coding for NADH/NADPH oxidase in male gender [95], interleukin-6 and stromelysin-1 in female gender [95], e-NOS in Caucasian and mostly in Asian populations [92], and ALDH (aldehyde dehydrogenase) activity [96]. ALDH2 deficiency, most common in the East Asian population, is strongly associated with CAS [97], with an increased effect due to the coexistence of smoking and/or alcohol [98, 99]. Furthermore, a strong association between ischemic heart disease and ALDH2 was found to be a powerful indicator of myocardial ischemic attacks [100, 101] and as a predictor for lower sensitivity to nitrate administration [102]. Eventually, in gene polymorphisms or mutations, we consider an environmental impact while no role has been identified for a family history.

### 5.8. Microvascular Dysfunction

The contributing role of microvascular dysfunction in the pathophysiology of INOCA is a current trend in cardiology. Female gender, low BMI (body mass index), decreased adenosine triphosphate-induced coronary flow reserve (ATP-CFR), borderline diastolic to systolic velocity ratio (DSVR), and minor ischemic ECG findings at rest are the identified predictors for microvascular CAS [103]. Microvascular dysfunction is characterized by a reduction in microvascular wall response to vasodilators in conjunction with hyperreactivity to vasoconstrictors. Furthermore, a significant coexistence of endothelial dysfunction in the systemic peripheral arterioles and in the coronary microcirculation was demonstrated in vasospastic angina [2].

## 6. Diagnosis

**Figure 1:**
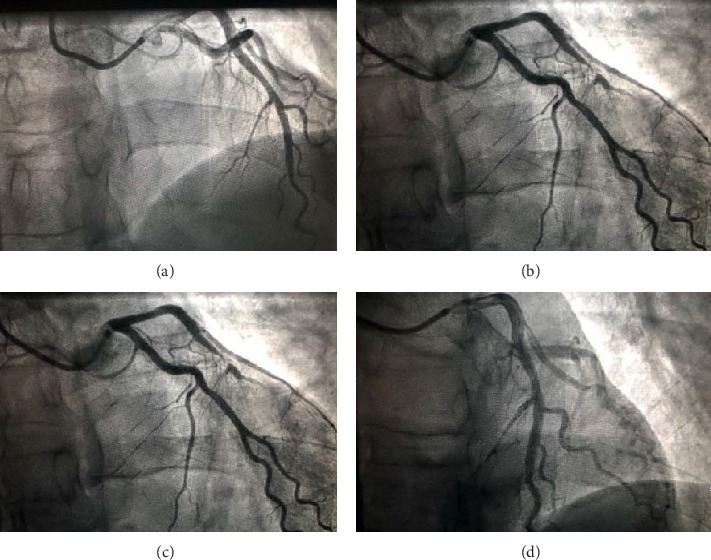
(a) Fluoroscopy showing a nonsignificant atheromatous lesion in the middle part of LAD in a 42-year-old patient with a familial history of sudden cardiac death. (b) Left coronary angiogram showing a subocclusive focal LAD coronary spasm after the methergin test. (c) Fluoroscopy showing an improved coronary blood flow after intracoronary nitrate injection. (d) Left coronary angiogram showing LAD returned to the basal blood flow. LAD : left anterior descending coronary artery.

The most reliable method for diagnosing CAS is the provocation test adjunctive to coronary angiography [104] with several vascular lumen reduction thresholds set at more than 50% [104], 70% [105], 75% [106], and 90% [5] or any luminal reduction associated to angina and/or ischemic electrocardiographic modifications [28]. Functional tests including intracoronary injection of ergonovine or acetylcholine are most commonly used [104] while intravenous administration was abandoned due to serious side effects [107–109].  Figure 1 shows a positive and reversible provocative test with subocclusion of the LAD. Intracoronary provocative testing can also lead to complications, especially when performed in multiple vessels, including angina, dyspnea, vomiting, and arrhythmias [28, 110]. It should not be performed in pregnant women and in patients with severe hypertension, significant left main stenosis, advanced heart failure, and severe aortic stenosis [104]. A false negative result is possible in case of low disease activity [111]. In addition, the vascular reactivity to nitroglycerin administration is tested by spasm provocation testing. Ohba et al. diagnosed microvascular CAS through detecting an increase in lactate production level in coronary circulation and a simultaneous decrease in coronary blood flow [103]. These measures were conducted on patients presenting for angina with no obstructive coronary artery disease in whom the intracoronary acetylcholine provocative test failed to show angiographic epicardial coronary spasm.

Current modalities of coronary imaging detect CAS-specific findings. For example, intravascular ultrasound study (IVUS) provides clues to differentiate vasospastic angina from nonvasospastic angina. It identifies a small lesion site plaque volume and burden and a diffuse coronary intimal thickening reflecting intimal hyperplasia in vasospastic angina [112] as well as different plaque components appearing as a hypoechoic, less-calcified, and fibrous-dominant plaque [113–115]. Therefore, IVUS studies contribute to understand the pathophysiology of CAS. IVUS detects the presence of occult atherosclerotic lesions at the site of focal coronary artery spasm even in the lack of angiographic disease [113–115]. This finding confirms the hypothesis that CAS is caused by local hypercontractility to vasoconstrictor stimulus on minimal atherosclerotic disease. Nevertheless, optical coherence tomography (OCT) delineates structural changes of coronary arteries in patients with CAS. In spastic coronaries, we find a reduced medial thickness reactivity and an increased medial area and thickness in comparison with normal coronaries [116]. Also, some relevant findings are revealed by OCT such as the presence of erosion at the coronary spasm site in one-third of cases and the presence of luminal irregularities in two-thirds of cases, suggesting the role of antiplatelet therapy in vasospastic angina [117].

Currently, cardiac magnetic resonance imaging (CMRI) is the gold standard, as it is safe and noninvasive for assessing MINOCA patients [118], but its role remains controversial in vasospastic angina [119]. Recent studies suggest, however, that a reduced myocardial blood flow or asynchronism in myocardial perfusion outlined by CMRI indicates CAS. The advantage of performing CMRI in MINOCA patients is eliminating other potential differential diagnoses such as takotsubo and myocarditis and then favoring a CAS attack [120, 121].

## 7. Treatment

Sublingual nitrate remains the main treatment to relieve acute attacks of CAS [122]. Smoking cessation, controlling risk factors, and avoiding precipitating factors are recommended to prevent CAS recurrences, especially in atherosclerotic cases [36]. The first therapeutic approach is based on long-acting calcium channel blockers (CCBs) preferably taken at night due to circadian variability of CAS [123, 124]. Benidipine had shown a better prognostic profile compared with other CCBs [125]. However, a near-complete reduction in CAS recurrence is observed with nondihydropyridine CCBs (e.g., diltiazem and verapamil) rather than dihydropyridine CCB [125]. Blocking calcium flow into the coronary artery smooth muscle and stimulating nitrate production to NO result in vascular vasodilation. No significant difference is demonstrated between the usage of CCBs or nitrate on reducing CAS recurrence [126]. The combination of dihydropyridine and nondihydropyridine CCBs may be used for severe symptoms [19]. Besides, CCBs act on potential CAS complications such as atrioventricular block representing an independent survival predictor [127]. Unless it is effective, regular use of nitrates is not recommended due to drug-related issues [128, 129]. Nicorandil, a potassium and nitrate channel activator, is recommended in CAS refractory to CCBs and nitrate [130]. It is worth mentioning that beta-blockers exacerbate CAS by enhancing a vasoconstrictive response [59] except for nebivolol which differs from other beta-blockers by its high beta-1 selectivity and its ability to produce NO promoting vasodilation [125]. Overall, studies have documented a positive role of statins in suppressing the CAS episode and decreasing the risk of recurrence via improving endothelial function [131, 132]. Also, the administration of magnesium and antioxidants such as vitamins C and E is helpful in the management of CAS [133, 134]. Aspirin has a controversial role: small doses result in vasodilation via inhibiting thromboxane A2, while large doses result in vasoconstriction via blocking prostacyclin production [135].

Several invasive approaches to drug refractory CAS were proposed. The feasibility and success rates of the coronary artery bypass graft are considered according to the underlying characteristics of CAS, as a focal, nondiffused atherosclerotic site of CAS is an essential determinant [136]. On the other hand, stent angioplasty is only recommended in patients with CAS and significant stenosis because the efficacy of coronary stenting to prevent vasospastic angina remains unclear in patients with nonobstructive lesions acknowledging that the recurrence of CAS to the stenting site was observed distally after intervention [137–140].

Finally, the indication for sympathectomy or implantable cardiac defibrillator was limited to life-threatening situations defined by cardiac arrest survivors and episodes of ventricular arrhythmia [141–143]. Despite that CAS can lead to lethal arrhythmias, ICD indication remains unclear and is based on each case situation on top of physician experience. To date, there are no recommendations for ICD implantation in primary prevention for CAS patients. In secondary prevention, current evidence shows a beneficial role of ICD in aborted sudden cardiac death CAS patients regardless of the optimal medical therapy [125]. Some report an appropriate ICD shock recorded in one-fourth of patients with failed sudden death, suggesting to use the result of the provocative pharmacological test performed under optimal medical therapy in resuscitated CAS patients as a determinant parameter for secondary ICD implantation [126]. Sympathetic denervation remains a promising therapeutic approach for severe refractory CAS. Only few cases with dramatic good outcome are reported in literature [144, 145].

## 8. Prognosis

The first 3 months following the CAS attack is the critical period with highest risk for cardiovascular events [146]. Smoking cessation and CCB therapy are the most determinant prognostic factors [147]. There is no clear evidence defining the appropriate duration to continue CCBs. Cardiac death occurs between 0 and 10% among patient with CAS [148, 149], and recurrent vasospastic angina was observed in 3.9 up to 18.6% [150, 151]. CAS in Japanese population is associated with better outcome than western population [151]. Advanced age, elevated Hs-CRP, multivessel spasm, significant atheromatous lesion, and reduced ejection fraction are poor prognostic predictors [151]. An increased rate of lethal arrhythmia and sudden cardiac death is detected [152]. Risk and prognostic stratification scores are elaborated by the Japanese Coronary Spasm Association in order to assess CAS patients [152].

## 9. Conclusion

CAS is a complex multifactorial disease that can lead to serious complications. A wide clinical spectrum is attributed, and sudden cardiac death may well be the uncovering event. As a result, an early recognition and appropriate medical approach is primordial. The prevalence of CAS tends do decrease with wide spread use of ACE inhibitors and statins and diminished performance of spasm provocation tests which are considered time consuming in highly active cardiac centers.

Although CMRI plays a key role in the differential diagnosis of MINOCA, further studies are needed to emphasize CMRI's utility in CAS diagnosis.

## Figures and Tables

**Table 1:** Pathophysiologic mechanisms.

Main pathophysiologic mechanisms in CAS
Autonomic nervous system
Endothelial dysfunction
Chronic inflammation
Oxidative stress
Smooth muscle hypercontractility
Atherosclerosis and thrombosis
Genetics

CAS : coronary artery spasm.
